# Lichenoid drug eruption induced by pravastatin; it is possible to prescribe other statins?^☆^

**DOI:** 10.1016/j.abd.2021.02.013

**Published:** 2022-11-01

**Authors:** Francisco J. Navarro-Triviño, Ricardo Ruiz-Villaverde

**Affiliations:** a Department of Contact Eczema and Immunoallergic Diseases, Dermatology, Hospital Universitario San Cecilio, Granada, Spain; b Department of Dermatology, Hospital Universitario San Cecilio; Instituto Biosanitario de Granada, Granada, Spain

Dear Editor,

Lichenoid drug eruptions are uncommon and may be difficult to differentiate from idiopathic lichen planus. Clinical and histopathological features are extremely similar in both diseases. Lichenoid drug eruptions caused by HMG-CoA reductase inhibitors are exceptional.

A 59-year-old caucasian man was attended to our outpatient dermatological clinic (Hospital Universitario San Cecilio, Granada, Spain) complaining of a 2-month history of pruritic eruption located on his chest and lumbar area. No previous history of any dermatological conditions was referred. His general practitioner prescribed Pravastatin/Fenofibrate (Pravafenix® 40/160 mg) for the treatment of hypercholesterolemia and hypertriglyceridemia 3 months ago. The patient was referred to our department with clinical suspicion of plaque psoriasis. Physical examination showed multiple shiny and violaceous erythematous-squamous plaques on the trunk and lumbar area (Fig. 1). Wickham’s striae could not be stated with dermoscopy. Mucosal examination showed no abnormalities. The histopathological study revealed a lichenoid inflammatory infiltrate with intense involvement of the dermo-epidermal interface associated with apoptotic keratinocytes and melanophages in the papillary dermis (Fig. 2). Lichenoid eruption due to pravastatin was then concluded. Pravastatin was discontinued and changed to fluvastatine. Fenofibrate was not discontinued. Topical corticosteroid treatment (mometasone furoate 0.1% cream, 1 day) was applied during the first seven days. Residual brownish hyperpigmentation was observed at 3 months of follow-up.

**Figure 1:**
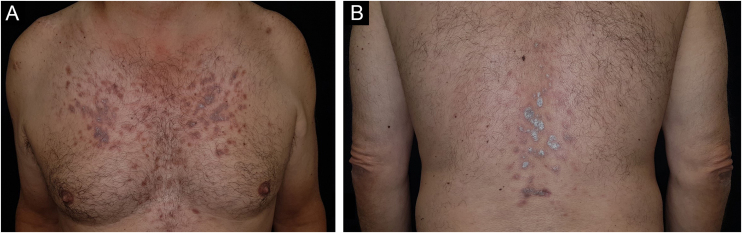
(A) multiples shiny and violaceous erythematous-squamous plaques on the trunk and (B) lumbar area.

**Figure 2:**
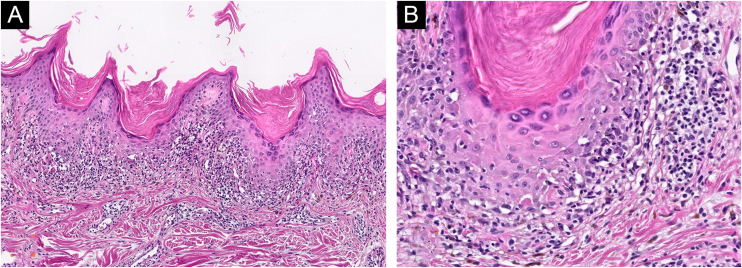
(A) Lichenoid inflammatory infiltrate with intense involvement of the dermo-epidermal interface (Hematoxylin & eosin, ×100). (B) Presence of apoptotic keratinocytes, eosinophils, focal parakeratosis, and melanophages in papillary dermis (Hematoxylin & eosin, ×200).

Statins are a commonly prescribed medication for the treatment of hypercholesterolemia. A limited number of case reports of lichenoid drug eruption associated with statins have been reported to date.^1^ The most frequently reported adverse dermatological reaction is an eczematous rash or hypersensitivity eruption. Atorvastatin, lovastatin, and simvastatin are the statins associated with the most adverse effects, including photosensitivity, urticaria, lupus-like syndrome, and pruritus, among others.^2^ The mechanism of statin-induced cutaneous eruptions is not well understood. The anti-inflammatory effects of statins could be related to both beneficial effects and the development of these side effects. The cell-mediated autoimmune reactions against basal layer keratinocytes are thought to be involved.^3^ Pravastatin does not undergo metabolism by CYP enzymes, unlike simvastatin, lovastatin, atorvastatin, and rosuvastatin. These drugs undergo their metabolism in the liver by cytochrome P450 CYP3A3 and CYP2C9 AV.^4^ This could possibly explain why our patient did not continue with the lichenoid eruption after starting fluvastatine. These metabolites could be related to the development of skin eruption. A few cases of lichenoid eruption induced by pravastatin have been reported.^5^ Moreover, psoriasis-like eczematous lesions have been reported with pravastatin.^6^ Both skin reactions tend to be resistant to topical corticosteroid treatments, and even to systemic treatments. Stopping the use of pravastatin will lead to the resolution of the clinical course. Although fluvastatine has been well tolerated by our patient, ezetimibe is considered a first-line therapy in the case of proven intolerance to statins.^7^ A case of recurrence of lichenoid eruption has been described in a patient who stopped rosuvastatin and was switched to simvastatin. The choice of a new therapeutic line should imply avoiding first-pass liver metabolism. Finally, our patient developed a score of 6 in the Naranjo algorithm, so the causal relationship was considered probable.

## Financial support

None declared.

## Author’s contribution

Francisco J. Navarro-Triviño: Approval of the final version of the manuscript; critical literature review; data collection, analysis and interpretation; effective participation in research orientation; intellectual participation in propaedeutic and/or therapeutic; management of studied cases; manuscript critical review; preparation and writing of the manuscript; statistical analysis; study conception and planning.

Ricardo Ruiz-Villaverde: Approval of the final version of the manuscript; critical literature review; data collection, analysis and interpretation; effective participation in research orientation; intellectual participation in propaedeutic and/or therapeutic; management of studied cases; manuscript critical review; preparation and writing of the manuscript; statistical analysis; study conception and planning.

## Conflicts of interest

None declared.
